# Supplementary material for: Preparation of Heavily Doped P-Type PbSe with High Thermoelectric Performance by the NaCl Salt-Assisted Approach
Source: Molecules. 2023 Mar 14;28(6):2629. doi: 10.3390/molecules28062629 (PMC10051061; doi:10.3390/molecules28062629)
Supplement: Supplementary file 1 [file molecules-28-02629-s001.zip › molecules-2232551-supplementary.pdf]

# Supporting Information

# Preparation of Heavily Doped P-Type PbSe with High Thermoelectric Performance by the NaCl Salt-Assisted Approach

**Xinru Ma [1], Xuxia Shai [1,*], Yu Ding [2,3,*], Jie Zheng [4], Jinsong Wang [5], Jiale Sun [1], Xiaorui Li [1], Weitao Chen [1], Tingting Wei [1], Weina Ren [1], Lei Gao [1], Shukang Deng [4,*] and Chunhua Zeng [1,*]**

[1] Faculty of Science, Institute of Physical and Engineering Science, Kunming University of Science and Technology, Kunming 650500, China
[2] College of Chemistry and Materials Science, Hubei Engineering University, Xiaogan 432000, China
[3] Nuode New Materials Co., Ltd., Shenzhen 518048, China
[4] Education Ministry Key Laboratory of Renewable Energy Advanced Materials and Manufacturing Technology, Yunnan Normal University, Kunming 650500, China
[5] Faculty of Materials Science and Engineering, Kunming University of Science and Technology, Kunming 650093, China
* Correspondence: xuxiashai@kust.edu.cn (X.S.); dy9802@126.com (Y.D.); skdeng@126.com (S.D.); chzeng83@kust.edu.cn (C.Z.)

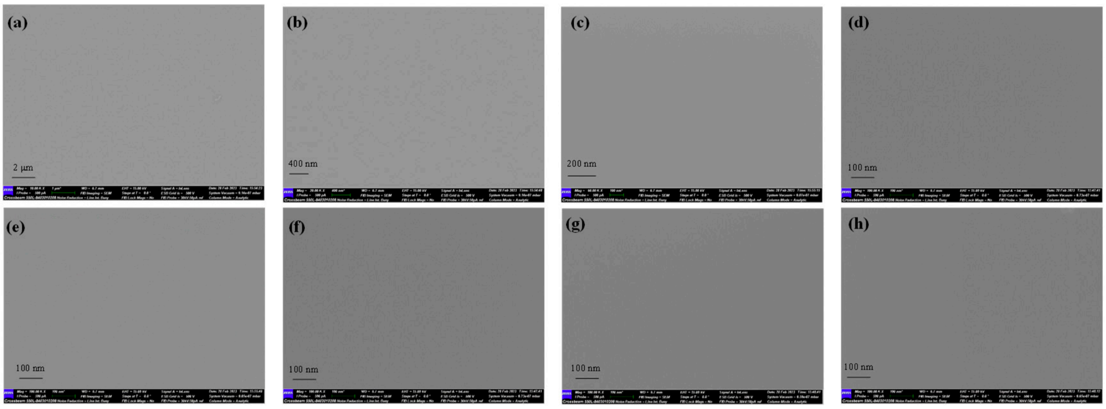


**Figure S1.** FESEM images of $PbSe(NaCl)_3$, which with different magnifications (10000-100000 magnification) (a-d) and from different positions at 100000 magnification (e-h).

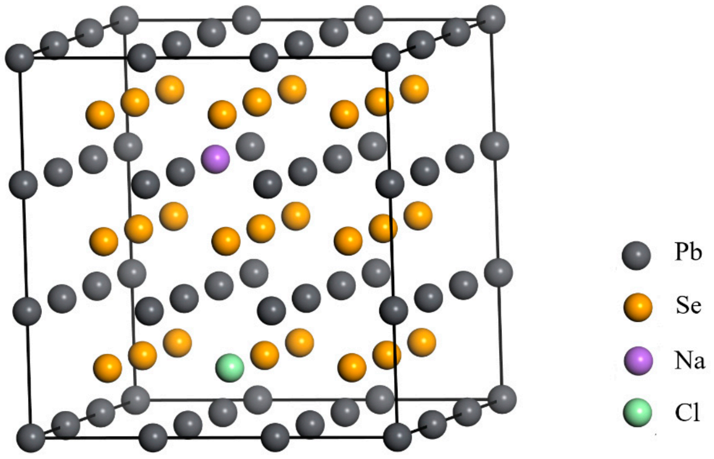


**Figure S2.** The crystal structure of PbSe doped with only one atom in each species.

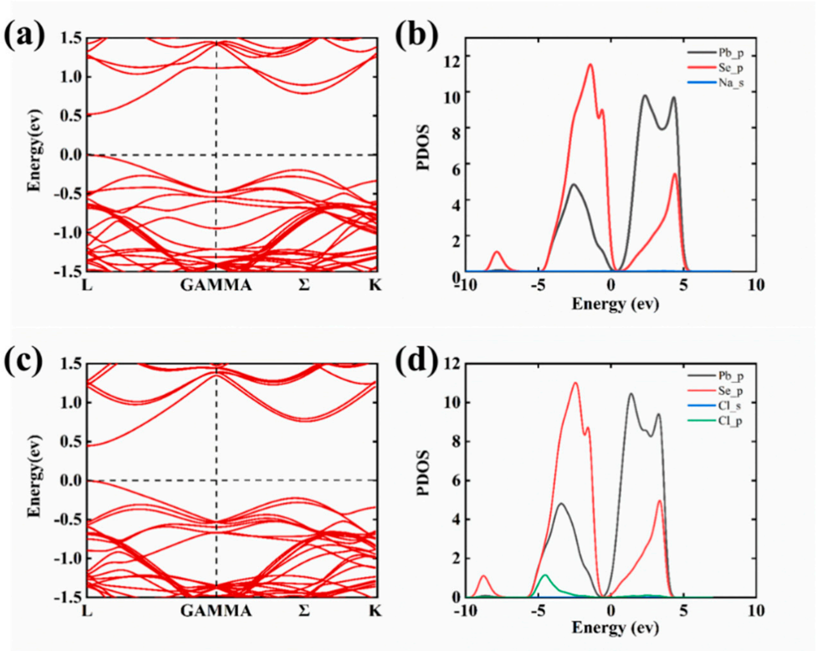


**Figure S3.** Band structure and DOS calculated using the 3×3×3 supercell. Band structure of Na-doped PbSe with ΔEv ~0.18 eV (a); Cl-doped PbSe with ΔEv ~0.22 eV (c); and corresponding density of states with only one atom substitution for Na-doped PbSe (b); and Cl-doped PbSe (d), where the corresponding states are displayed to clearly show their contribution.

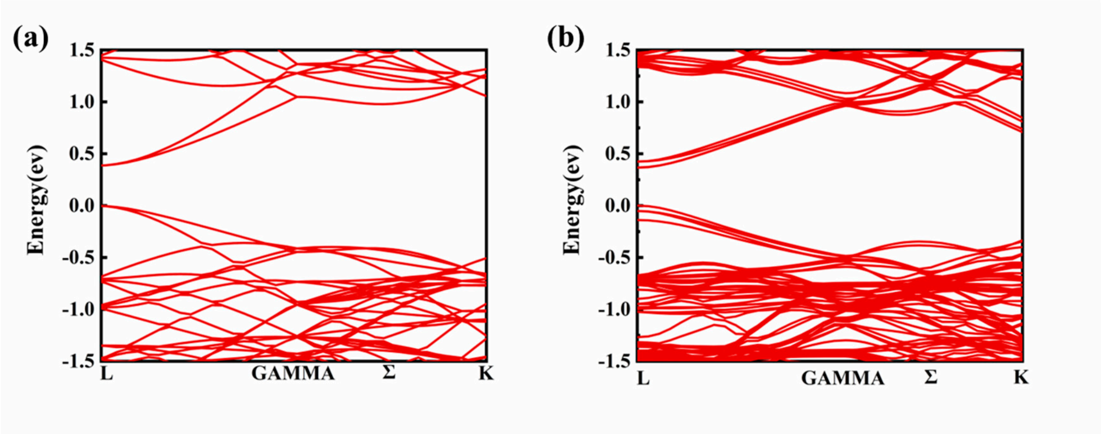


**Figure S4.** Band structure calculated using the 4×4×4 supercell. Band structure of PbSe with ΔEv ~0.42 eV (a); NaCl-doped PbSe with ΔEv ~0.33 eV (b).
